# Culturally Tailored Messages and Trial Registry Enrollment: A Randomized Clinical Trial

**DOI:** 10.1001/jamanetworkopen.2024.44229

**Published:** 2024-11-12

**Authors:** Kevin B. Johnson, Stacy L. Iannone, Susan L. Furth, Lynne Taylor, Andy S. L. Tan

**Affiliations:** 1 Division of Biomedical Informatics, Department of Biostatistics, Epidemiology, and Informatics, Perelman School of Medicine, University of Pennsylvania, Children’s Hospital of Philadelphia, Philadelphia; 2 The Annenberg School for Communication, University of Pennsylvania, Philadelphia; 3 Leonard Davis Institute of Health Economics, University of Pennsylvania, Philadelphia; 4 Department of Biostatistics, Epidemiology, and Informatics, Perelman School of Medicine, University of Pennsylvania, Philadelphia; 5 Pediatrics Nephrology, Perelman School of Medicine, University of Pennsylvania, Philadelphia; 6 Pediatric Research, Children’s Hospital of Philadelphia, Philadelphia, Pennsylvania; 7 Center for Clinical Epidemiology and Biostatistics, Biostatistics Analysis Center, Perelman School of Medicine, University of Pennsylvania, Philadelphia; 8 Penn Medicine Abramson Cancer Center, Tobacco and Environmental Carcinogenesis Program, Philadelphia, Pennsylvania

## Abstract

**Importance:**

Marginalized populations have lower levels of clinical trial representation than other populations. Tailoring recruitment materials and providing incentives may improve representation.

**Objective:**

To determine whether culturally tailored video improves parents’ decision to enroll (DTE) Black children in a research registry.

**Design, Setting, and Participants:**

A randomized clinical trial, using mixed methods and consecutive sampling, was conducted in an urban primary care pediatric clinic. A post hoc study assessed the association of a monetary incentive with DTE. Ninety-three parents were recruited before their child’s scheduled clinic visit. Participants completed a survey during which they were randomly assigned to review a paragraph (control group), generic video, or tailored video and received a gift card. Video watchers completed questions about its effectiveness. All participants were invited to join the registry. In the post hoc study, consenting participants followed the control group protocol and the coordinator promised them an ink pen for participating. The study was conducted between November 15, 2022, and August 29, 2023.

**Intervention:**

A survey regarding the importance of being included in research studies was administered after participants watched a culturally tailored video.

**Main Outcome and Measures:**

The primary outcome was DTE. Covariates were message effectiveness, clinical trial knowledge, and trust. Intention-to-treat analysis was performed.

**Results:**

A total of 125 individuals were included. All groups had similar mean (SD) parent age (overall, 32.6 [7.35] years), gender (110 [88.0%] women), and race (116 [92.8%] Black or African American). There was no significant difference in the odds of DTE between the appeal video and control (odds ratio [OR], 1.19; 95% CI, 0.38-3.72) or between the generic video and control (OR, 1.91; 95% CI, 0.54-6.71). However, DTE was higher for all 3 gift card groups vs the ink pen incentive group (eg, control vs ink pen incentive: OR, 5.01; 95% CI, 1.68-14.95). After adjusting for trust, monetary incentives were associated with higher odds of DTE (adjusted OR, 5.92; 95% CI, 2.44-14.39).

**Conclusions and Relevance:**

In this randomized clinical trial, targeted messaging was not associated with a higher decision to enroll in a pediatric clinical trial registry. There may be a role for combining holistic messaging with monetary incentives to improve enrollment.

**Trial Registration:**

ClinicalTrials.gov Identifier: NCT06138145

## Key Points

**Question:**

Does a culturally tailored video message impact parents’ willingness to enroll Black children in clinical trials?

**Findings:**

In this randomized clinical trial including 125 individuals comparing registry enrollment rates after seeing a generic video, culturally tailored video, or no video with or without a monetary incentive to participate: all messages had similar enrollment rates (72%)—lower when receiving an ink pen incentive (34%). After adjusting for trust, receiving a monetary incentive was associated with higher odds of enrollment.

**Meaning:**

The findings of this trial suggest targeted messaging is not associated with a higher decision to enroll in a pediatric clinical trial registry; combining messaging with monetary incentives may improve clinical trials enrollment.

## Teaser

This randomized clinical trial examines the use of a culturally tailored video for Black parents as a messaging tool to increase enrollment in pediatric clinical trials.

## Introduction

Children from racially and ethnically marginalized groups in the US are less likely to be enrolled in randomized clinical trials than other populations, placing these groups at an increased risk for diagnostic and treatment disparities for innovative approaches to care. Historical data about the enrollment of Black or African American individuals is likely impacted by potential unwillingness to disclose race and ethnicity (underreporting bias).^1^ One study suggested that with increasing reporting, the lower-than-expected enrollment of marginalized children persists.^2^ Although there are myriad reasons for this, one potentially modifiable reason is health care system trust among these groups.^3,4^

Adult studies have reported variable success in overcoming historical mistrust using culturally tailored messaging.^5,6,7,8^ Cultural tailoring, defined as adapting messages to the cultural characteristics of an ethnic group,^9^ may overcome known obstacles, such as having only White researchers deliver messages to minoritized individuals^10^ or not considering the cultural, historical, and linguistic differences between groups. Although surface tailoring by changing the face of the message delivered is straightforward and occasionally useful,^7^ the literature better supports tailoring to social and historical characteristics, values, and traditions.^8,11,12,13^ There are limited data about the potential for cultural tailoring in pediatrics. Zhu and colleagues^14^ demonstrated some benefit of altruism-eliciting (inclusion) appeal messaging on COVID-19 vaccine uptake among youth with neutral opinions. Stehr and colleagues^15^ systematically examined communication strategies and observed that tailoring could contribute to possibly effective communication.

An alternative to circumventing mistrust through cultural tailoring may be to respond more directly to the historical and ongoing perceived abuses to marginalized communities by health systems. From that perspective, tailoring alone may be insufficient; behavioral economic techniques, such as public recognition, charitable donations,^16^ nudges,^17^ or direct financial incentives may be effective.^18^ Financial incentives have had variable success in overcoming obstacles to pediatric and younger adult behavior change.^19,20^

The objectives of this project were to codesign culturally tailored inclusion appeals for parents of younger children and evaluate the effects of these appeals vs standard recruitment strategy. The study hypothesized that culturally tailored videos codesigned with community advisors and including appeals from Black families would increase their decision to enroll (DTE) rate in clinical trials compared with a generic video or written information about trial enrollment (H1). We further hypothesized that high baseline levels of trust and clinical trial knowledge would be associated with higher DTE (H2). This study further tested the hypothesis that financial incentives to enroll would be associated with higher DTE (H3).

## Methods

### Study Sample

The Children’s Hospital of Pennsylvania (CHOP) Institutional Review Board approved the study, confirming adherence to ethical standards and regulatory requirements before starting the research. The clinical trial protocol is available in Supplement 1. Participants provided informed consent and were told they would receive a $25 gift card. The study was considered a pilot study because of our decision to focus on families who identified as Black or African American, with an understanding that these results would lead to a larger study focusing on more historically disadvantaged groups, and how tailored messaging impacts historically disadvantaged group enrollment in a clinical trial. This report followed the Consolidated Standards of Reporting Trials (CONSORT) reporting guideline for randomized studies.

We invited US younger adults with children aged 0 to 12 years being seen in primary clinics in Philadelphia to complete a survey about joining a pediatric research registry. A research coordinator recruited parents between November 15, 2022, and August 29, 2023, focusing on 2 clinics with a large percentage of Black patients, as assessed by trained research team members and based on self-reported data in our scheduling system. The research coordinator obtained written informed consent as the first question in the survey. We also worked with a subset of the CHOP Research Advisory Group (RAG).

### Mixed Methods Approach to Video Development

We worked with a subset of the CHOP RAG to create a culturally tailored video, which was designed to appeal to Black families about the importance of being included in research studies. The RAG provided input to create the video, including reviewing the script and selecting a filming location. The RAG includes family members of CHOP patients who, in addition to advising CHOP leadership, consult on research projects involving CHOP patients or families. The RAG voted on the aspects of cultural tailoring that would be important for the message, including social and historical characteristics. They were asked what would be relevant to the community based on constructs from the health belief model.^21^ They determined that risk susceptibility, severity, and action benefits would most likely be relevant to the community. Once we had a set of candidate appeals based on these constructs, the RAG reviewed and evaluated each regarding message targetedness and possible effectiveness. Based on their feedback, we created the appeal video for the study. Separately, the CHOP research registry team designed and produced a video describing the research registry and encouraging parents to join. This video, hereinafter called the generic video, featured a professional speaker who described the registry over static pictures of the hospital, research laboratories, and children, followed by an approximately 1-minute appeal by a physician to all families to join the registry.

For our appeal videos, we edited the generic video to include a targeted appeal using both rational messaging (ie, “...if it wasn’t for Brown and Black families participating in research, results of these studies might not benefit people like us.”) and bandwagon messaging (ie, “We’re parents here at CHOP. We’ve also been a part of the research going on here. And we need you to join us.”) The inclusion appeal video further acknowledged that there are historical reasons why Black or African American parents may mistrust medical institutions and be hesitant to enroll in clinical research to address tailoring to social and historical characteristics (ie, “In this country there has been a history of research impacting Black and Brown families in a negative way.”) These segments replaced other video segments of the same length to ensure that all videos were approximately the same duration. The generic appeal^22^ and the appeal with mother and daughter^23^ videos have been posted on a hosting site.

### Study Protocol

The Figure summarizes the trial flow profile. Participants completed the study using a web-based survey tool (Qualtrics XM; Qualtrics). Participants were first screened for eligibility. We included individuals who identified as parents or guardians of children younger than 13 years. Eligible participants completed demographics questions, a 3-question section about clinical trials knowledge, and a 5-question section about health information trust from the National Institutes of Health Health Information National Trends Survey (HINTS).^24^ They were then randomly assigned using the built-in randomizer function of the survey tool to (1) control (read information about the registry), (2) generic (view a generic video about the registry), or (3) appeal (like the generic video, but edited to include the culturally tailored video segment) groups. Participants who were randomized to view either video completed a 6-item perceived message effectiveness scale after viewing.^25^ All participants answered 1 final question to determine whether they would like to be enrolled in the registry. If they agreed to enroll, a research assistant helped them with the enrollment process.

**Figure.**
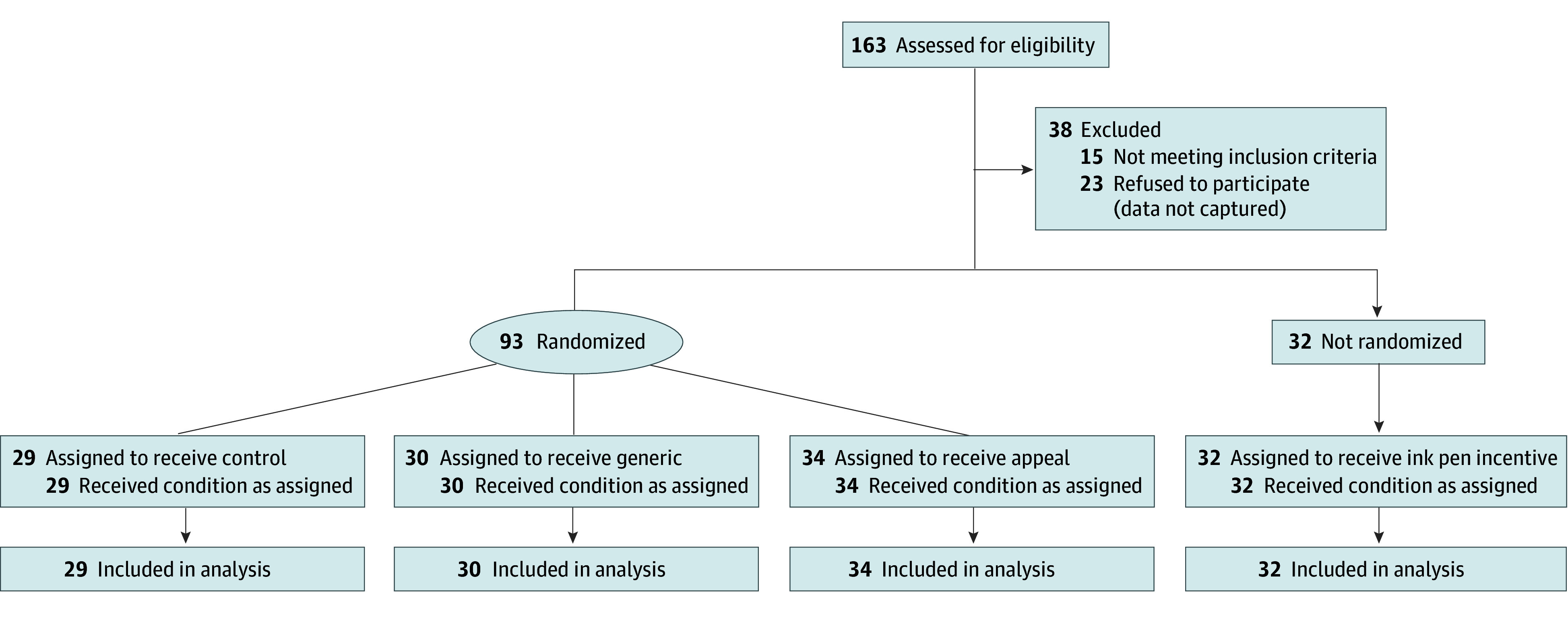
Profile of the Randomized Clinical Trial and Post Hoc Analysis

### Post Hoc Study

Preliminary information from our onsite research coordinator suggested that most parents were deciding to enroll in the registry. We sought to examine the influence of the $25 gift card on the decision to enroll. For that reason, we performed a post hoc study in the same settings as the trial, where we repeated the control condition (being presented only with information to read about the registry) but with a revised invitation to participate and promise of an ink pen as opposed to a gift card. This group was designated as the ink pen group. This process resulted in 4 groups: (1) control (text introduction from the registry, with a gift card), (2) generic (video detailing the registry’s objectives, no tailoring, with a gift card), (3) appeal (generic video edited to include a tailored appeal, with a gift card), and (4) ink pen (similar to control but received a university ink pen).

### Measures

The primary outcome was the decision to enroll, measured as yes or no. Covariates included clinical trials knowledge, health information trust, perceived message effectiveness, and race and ethnicity. We used a 2-item scale from the HINTS^19^ to assess clinical trials knowledge. Questions included previous participation (yes or no), level of knowledge (0, 1, 2, 3), and potential influence of altruism on their decision to participate in a trial (0, 1, 2, 3). The total possible scores ranged from 0 to 3, with higher scores indicating more knowledge.

For health information trust, we used a 5-item, 4-point ordinal scale from HINTS (0 = not at all to 3 = a lot).^24^ The survey item investigates the level of trust placed in 5 sources of health information: physicians, family or friends, government health agencies, religious organizations, and health groups. The item sum indicates the level of trust and yields a score span of 0 to 15, where higher scores indicate more trust. The Cronbach α level was 0.61, indicating the items measured different discernible domains or components of hospital trust.

To measure perceived message effectiveness, we used a 6-item scale based on work done by Davis and colleagues.^25^ The perceived message effectiveness score was the sum of the 6 items, yielding a total possible score of 6 to 30, with higher scores indicating higher message effectiveness (Cronbach α = 0.91). These measures were collected from participants who viewed the generic or appeal video.

We assessed race and ethnicity using language derived from recent National Institutes of Health guidance on collecting these data.^26^ We included this demographic variable to understand whether our targeted strategy to focus on Black families was achieved for this study of culturally tailored video messages.

### Statistical Analysis

The study-dependent variable was the decision to enroll in the research registry (DTE) as a function of the message type viewed by the participant. We calculated the sample size assuming an effect size of 0.4, based on data published by Skinner and colleagues^6^ for a 2-arm study involving ethnic minorities, where a tailored message increased participation from 43% to 62%. We determined that a final sample size of 25 patients per arm would be sufficient based on this effect size. We factored in a 10% attrition rate among participants.

We used graphs and summary statistics (percentages, mean, median, kurtosis, skewness) to examine the distribution of covariate and outcome variables, describe the sample, and help determine the appropriate analysis for each hypothesis. All analyses followed the intention-to-treat analytic approach. To examine group equality on demographic variables, baseline level of trust, and clinical trial knowledge, we used χ^2^ analysis for categorical variables and general linear model analysis of variance for interval-level variables (Supplement 1). We calculated *P* values, with *P* < .05 indicating statistical significance.

To examine messaging type and DTE (H1: the 3-arm randomized clinical trial study), we conducted a χ^2^ analysis and logistic regression. Using logistic regression, we modeled 1 = enrollment and used maximum likelihood model estimates to compute the DTE odds ratio (OR) and 95% Wald CIs. To assess the level of trust and DTE and clinical trials knowledge and DTE (H2), we computed separate rank biserial correlations for each incentivized group (text, generic, and appeal) and the nonrandomized ink pen messaging group.

To assess our secondary hypothesis that a financial incentive of $25 to enroll in the study would lead to higher DTE vs being offered an ink pen (H3), we computed the ORs between the nonrandomized ink pen incentive group and each randomized gift card incentivized group. All analyses were conducted using SAS Statistical software, version 15.1 (SAS Institute Inc).

## Results

Table 1 summarizes the characteristics of the overall sample. A total of 125 participants were included in the analysis (29 in the control group, 30 in the generic group, 34 in the appeal group, and 32 in the ink pen incentive group; 110 [88.0%] were women; 15 [12.0%] were men; mean [SD] age, 32.6 [7.35] years), and 116 (92.8%) self-identified as Black or African American. Seven participants (5.6%) had not completed high school. Of 93 randomized children, 47 (50.5%) were male and 46 (49.5%) were female. The group receiving the ink pen incentive had a significantly lower baseline level of trust than the group watching the generic video (mean [SD], 8.9 [2.58] vs 10.7 [2.55]; *P* = .03) but was otherwise similar to the other groups. Clinical trials knowledge was similar for all groups (0.88 [0.76] of 3 points; *P* = .54).

**Table 1.** Characteristics of the Intervention Groups

Characteristic	Overall (N = 125)	Intervention group, No. (%)			
		Control (n = 29)	Generic (n = 30)	Appeal (n = 34)	Ink pen incentive (n = 32)
**Demographic covariate**					
Parent age, mean (SD), y	NA	32.3 (7.94)	32.8 (6.88)	32.3 (6.34)	33.1 (8.45)
Sex					
Female	110 (88.0)	26 (89.7)	26 (86.7)	30 (88.2)	28 (87.5)
Male	15 (12.0)	3 (10.3)	4 (13.3)	4 (11.8)	4 (12.5)
Race and ethnicity					
Black	116 (92.8)	28 (96.7)	28 (93.3)	30 (88.2)	30 (93.8)
Hispanic	5 (4.0)	0	0	3 (8.8)	2 (6.3)
Not Hispanic	120 (96.0)	29 (100)	30 (100)	31 (91.2)	30 (93.8)
Other^a^	9 (7.2)	1 (3.5)	2 (6.7)	4 (11.8)	2 (6.3)
Parental educational level					
Less than high school	7 (5.6)	2 (6.9)	1 (3.3)	3 (8.8)	1 (3)
High school degree/equivalent	75 (60.5)	20 (69)	17 (57)	19 (56)	19 (59)
Associate degree	20 (16.1)	1 (3.5)	5 (17)	7 (21)	7 (22)
Bachelor’s, Master’s, professional degree	22 (17.8)	6 (21)	7 (23)	5 (15)	5 (16)
Decision to enroll, yes	82 (66.1)	21 (72.4)	25 (83.3)	25 (75.8)	11 (34.4)^b^
**Mediating covariate**					
Level of trust (range, 0-15), mean (SD)	9.6 (2.4)	9.3 (2.0)	10.7 (2.6)^c^	9.7 (2.3)	8.9 (2.6)^c^
Clinical trials knowledge (range, 0-3), mean (SD)	0.88 (0.76)	0.83 (0.80)	1.00 (0.69)	0.94 (0.74)	0.81 (0.78)

Abbreviation: NA, not applicable.

^a^ Includes Asian (n = 1); Native American/Alaska Native (n = 1), White (n = 3), and Other (n = 4).

^b^ *P* < .001.

^c^ *P* = .03.

### H1: Association Between Messaging Type DTE

Table 1 and Table 2 summarize the trial results. The overall rate of DTE was 65.6%. The generic video group had the highest DTE rate (83.3%), followed by the appeal video (75.8%). The odds of DTE were not significantly different between the 3 types of messaging: appeal video vs control (OR, 1.19; 95% CI, 0.38-3.72), generic video vs control (OR, 1.91; 95% CI, 0.54-6.71), and generic vs appeal (OR, 0.63; 955 CI, 0.18-2.18). Both the generic video and the appeal video had similar scores for message effectiveness (mean [SD] of a possible 30 points, 22.5 [7.1] vs 24.2 [4.6]; *P* = .25).

**Table 2.** Group Enrollment and the Effect of Message Type on the Decision to Enroll

Covariate	Unadjusted odds ratio (95% CI)
**Analysis**	
Appeal vs control	1.19 (0.38-3.72)
Appeal vs generic	0.63 (0.18-2.18)
Generic vs control	1.91 (0.54-6.71)
**Financial incentive comparisons**	
Appeal vs ink pen incentive	5.97 (2.03-17.56)
Control vs ink pen incentive	5.01 (1.68-14.95)
Generic vs ink pen incentive	9.55 (2.86-31.88)

### H2: Association Between Trust and Clinical Trials Knowledge of DTE

We examined the association between trust and knowledge and DTE within each group. In the ink pen group, higher trust was associated with DTE (n = 32; *r* = 0.38; *P* = .03). In contrast, in the gift card groups, trust had no association with DTE in the overall (*r* = 0.03; *P* = .75), appeal (n = 33, *r* = 0; *P* = .98), control (n = 29; *r* = 0.21; *P* = .28), and generic (n = 30; *r* = −0.23; *P* = .21) analyses. Clinical trials knowledge was associated with DTE (OR, 2.05; 95% CI, 1.16-3.63).

### H3: Role of Financial Incentives

The ink pen group had the lowest DTE (34.4%). The odds of DTE were significantly higher for each gift card group in comparison with the ink pen incentive group. Specifically, the appeal group had a 6 times higher odds of DTE compared with the ink pen incentive group (OR, 5.97; 95% CI, 2.03-17.56), while the control group had a 5 times higher odds of DTE in comparison with the ink pen incentive group (OR, 5.01; 95% CI, 1.68-14.95). More notably, the generic group had a 9 times higher odds of DTE in contrast to the ink pen group (OR, 9.55; 95% CI, 2.9-31.9). Clinical trials knowledge was associated with DTE in the ink pen group (n = 32; *r* = 0.40; *P* = .02) in contrast to the negligible correlations in the gift card groups (incentivized overall: n = 92; *r* = 0.13; *P* = .22; appeal: n = 33; *r* = 0.07; *P* = .69; control: n = 29; *r* = 0.15; *P* = .42 ; and generic: n = 30; *r* = 0.12; *P* = .49). After adjusting for trust, monetary incentives were associated with higher odds of DTE (adjusted OR, 5.92; 95% CI, 2.44-14.39).

## Discussion

Our study examined whether baseline trust and clinical trial knowledge levels could be increased with progressively more tailored messaging and monetary incentives. We did not observe a significant incremental benefit of replacing the text information with video (either generic or culturally tailored) on increasing DTE when a financial incentive was provided. Instead, we observed that all 3 groups receiving a gift card for reviewing text, generic video, or tailored video had significantly higher DTE than the group receiving text information and an ink pen. The reason may be offering a modest incentive (a $25 gift card) outweighed potential effects of the type of message (text, generic video, or tailored video) on DTE.

We found that the association between trust and DTE changes, with trust being an important consideration when there is no incentive but less relevant when there is an incentive. Our data suggest that monetary incentives alone may be more effective in changing behavior in this population than using targeted videos alone. This finding may be related to baseline levels of mistrust that even targeted videos cannot reverse, but that may be overcome using monetary incentives. However, one must ensure that the level of monetary incentives is not coercive, is commensurate with the level of risk and demands on participants’ time, and is reviewed by ethics review boards to protect participants’ autonomy. Other studies focusing on using only culturally tailored messages have yielded minimal improvement in outcomes, especially in light of the effort required to tailor messages for even 1 subpopulation of interest.^7,27^

The literature on the acceptability of direct financial incentives is mixed. Hoskins and colleagues^20^ conducted a systematic review to understand the evidence of the acceptability of using financial incentives for health-related behavior change. Their findings note that fairness, messaging, character, liberty, and trade-offs may determine whether this approach should be acceptable. Other research, such as by de Keyser and colleagues,^19^ has explored this approach in pediatrics based on feasibility and acceptability studies with variable success and no apparent downside.

### Strengths and Limitations

One strength in our study is emphasizing parents’ voices during the research and intervention design process and integrating their decisions in implementing the intervention trial. We used mixed methods research, beginning with a focus group of patient’s families, known as the RAG, who provided study design advice, codesigned the video content, iteratively refined the script for the videos, and selected the final culturally tailored appeal used in the trial.

These results are subject to study limitations. First, this study sampled patients from only a few sites in 1 primarily urban setting. Other marginalized groups were underrepresented for these findings to be generalizable. Although similar to the 3 randomized arms, participants in the fourth arm of the study differed in terms of baseline trust and the timing for data collection. This arm was recruited approximately 6 months after the other 3. National or institutional activities could have negatively impacted trust during that time, which might have set a different level of receptivity to deciding to participate in clinical trials. Patients were aware of the incentive before they agreed to participate; therefore, baseline trust will represent both their a priori beliefs and an update they may have had once the study team mentioned the incentive.

Our study used only baseline, self-reported assessments of trust and clinical trials knowledge, with no additional data collected after watching the videos or receiving news about the incentive. Therefore, we cannot assert how trust and clinical trials knowledge changed or whether DTE was attributable to that change.

In addition, this was a pilot trial because it attempted to assess the impact of culturally tailored videos for only 1 population. Based on these results, it will be important to design and conduct a larger trial involving more historically marginalized populations, different incentive models, and perhaps using trial enrollment, rather than DTE in a registry, as the primary outcome.

## Conclusions

The data from this randomized clinical trial suggest a potential role for combining holistic messaging with monetary incentives to overcome preconceived mistrust or lack of knowledge about the importance of clinical trials. These data also suggest that, in the presence of monetary incentives, cultural tailoring may have less impact on patient decision-making than previously considered. Further research is needed to examine whether monetary incentives overcome prior mistrust and knowledge deficits.

## Notes

Supplement 1.
Trial Protocol and Statistical Analysis Plan

Supplement 2.
Data Sharing Statement
