# Supplementary material for: Culturally Tailored Messages and Trial Registry Enrollment: A Randomized Clinical Trial
Source: JAMA Netw Open. 2024 Nov 12;7(11):e2444229. doi: 10.1001/jamanetworkopen.2024.44229 (PMC11558476; doi:10.1001/jamanetworkopen.2024.44229)
Supplement: Supplement 1. — Trial Protocol and Statistical Analysis Plan [file jamanetwopen-e2444229-s001.pdf]

Title: Testing the Impact of Appeals to Diversity and Inclusion in Pediatric Clinical Research Study Enrollment Videos: A Randomized Pilot Study

Short Title: Inclusion Appeals and Enrollment Equity

Study Principal Investigator(s)

Kevin B. Johnson, MD MS FAAP FAMIA FACMI

B202 Richards Building

3700 Hamilton Walk

University of Pennsylvania | Philadelphia, PA 19104-6116

215-573-5885

Kevin.johnson1@pennmedicine.upenn.edu

Andy SL Tan, PhD MPH MBA MBBS

375 Longwood Ave LW662, Boston, MA 02215

Andy.tan@asc.upenn.edu

ABSTRACT

Context:

African and African American individuals are inadequately represented in clinical trials data—impacting the relevance of these trials to diseases that affect them. Although studies have demonstrated the value of video, little is known about the value of incorporating appeals to promote inclusion.

Objectives:

This study will assess whether video-based inclusion appeals improve African American children's enrollment into the Children's Hospital of Philadelphia (CHOP) research study registry.

Study Design:

The study will consist of two phases: a qualitative research phase and a randomized controlled trial phase.

Setting/Participants:

Phase 1 involves parents from the CHOP Research Family Partners and utilizes consensus/voting to review study design and materials. Phase 2 takes place in the CHOP primary care waiting rooms. Ninety parents who self-identify as English-speaking African/African American adults will be enrolled.

Study Interventions and Measures:

Phase 1 has been described above. Phase 2 study participants will complete a demographic survey, before being randomized to receive one of three interventions:

1. Short description of the registry
2. video with inclusion appeal, questions about the perceived effectiveness of the video
3. video without inclusion appeal, questions about the perceived effectiveness of the video.

All participants will be invited to join a study enrollment registry.

SCHEDULE OF STUDY PROCEDURES

**Study Phase 1**	**Group Meeting**	**Group Meeting**	**Group Meeting**	**Group Meeting**
**Visit Number**	**1**	**2**	**3**	**4**
Informed Consent/Assent	X			
Project discussion	X	X	X	X
Video review		X	X	
Video discussion		X	X	
Video evaluation		X	X	
Enrollment plan	X			X

**Study Phase 2**	**Screening**	**Intervention**
**Visit Number**	**1**	**1**
**Study Days**	**1**	
Informed Consent/Assent	X	
Review Inclusion/Exclusion Criteria	X	
Randomization	X	
Demographics/Medical History		X
Survey (video embedded if being shown)		X
Registry enrollment question		X

STUDY DIAGRAM

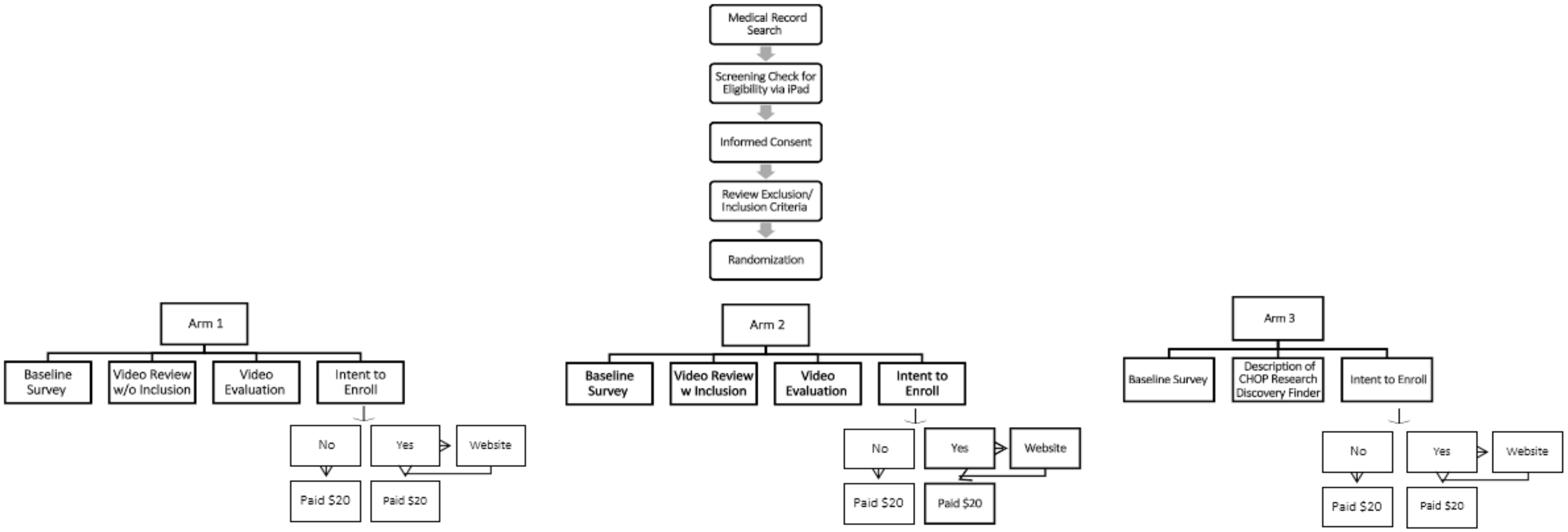

1 BACKGROUND INFORMATION AND RATIONALE

1.1 Introduction

Improving diversity in research projects and national data repositories is critical to providing safe and effective care to marginalized groups. Unfortunately, certain groups, such as African Americans, are inadequately represented in much of the available data and in current clinical trials. Recently, investigators have begun employing new messaging approaches to increase diversity in clinical trial enrollment. These messages are typically designed to inform, with no appeal for specific groups to act. Very little is known about the potential impact of inclusion appeals--direct appeals to historically marginalized groups calling on their need to be included in research studies. Applying the science of appeals to recruitment messages may improve the effectiveness of educational videos and lead to greater participation in research. This pilot study proposes to empirically study whether an inclusion appeal increases attempts to enroll children in studies.

1.2 Name and Description of Investigational Product or Intervention

The intervention will be a short video segment added to an informational video about Pediatric Clinical Research Study that will be tailored to appeal to the need for an inclusive population of study participants in clinical trials. For this pilot study, we will target African/African American participants.

1.3 Findings from Non-Clinical and Clinical Studies

n/a

1.4 Relevant Literature and Data

Recently, investigators have begun employing new messaging approaches to increase diversity in clinical trial enrollment. For example, transportation theory-based messaging (immersion into a narrative world)[1] and video narratives[2] have demonstrated an impact on persuasion, while in the pediatric literature, video messaging has been used to improve parental participation in reading to children[3],In pediatric studies, video enrollment messaging has had mixed results in terms of impacting marginalized groups[4] [5]. In our review of the literature, while these studies employ video messaging, investigators have not provided evidence that they applied *the science* of science communication to constructing

---

[1] Melanie C. Green and Timothy C. Brock, "The Role of Transportation in the Persuasiveness of Public Narratives.," *Journal of Personality and Social Psychology* 79, no. 5 (November 2000): 701–21, https://doi.org/10.1037/0022-3514.79.5.701.
[2] Fuyuan Shen, Vivian C. Sheer, and Ruobing Li, "Impact of Narratives on Persuasion in Health Communication: A Meta-Analysis," *Journal of Advertising* 44, no. 2 (April 3, 2015): 105–13, https://doi.org/10.1080/00913367.2015.1018467.
[3] Manuel E. Jimenez et al., "Enhancing Reach Out and Read With a Video and Text Messages: A Randomized Trial in a Low-Income Predominantly Latino Sample," *Academic Pediatrics* 21, no. 6 (August 2021): 968–76, https://doi.org/10.1016/j.acap.2021.02.011.
[4] Jimenez et al.
[5] Paula M. Frew et al., "A Randomized Trial of Maternal Influenza Immunization Decision-Making: A Test of Persuasive Messaging Models," *Human Vaccines & Immunotherapeutics* 12, no. 8 (August 2, 2016): 1989–96, https://doi.org/10.1080/21645515.2016.1199309.

these videos and did not publish any evaluation of the message's effectiveness at conveying its intended message. For example, a recent study by Skinner and colleagues[6] investigated the role of an educational video on clinical trial enrollment among ethnic minorities. It showed no effect; however, it utilized a DVD-based intervention that was only watched by half the participants, which the authors acknowledged as a limitation. Finally, none of these projects appear to have included any sort of message that specifically appealed to a target audience. *Appeals* are a well-established approach in communication that utilize evidence or emotional triggers to persuade the receiver to change his or her behavior.[7][8][9]

1.5 Compliance Statement

This study will be conducted in full accordance all applicable Children's Hospital of Philadelphia Research Policies and Procedures and all applicable Federal and state laws and regulations including 45 CFR 46, 21 CFR Parts 50, 54, 56, 312, 314 and 812 and the Good Clinical Practice: Consolidated Guideline approved by the International Conference on Harmonisation (ICH). All episodes of noncompliance will be documented.

The investigators will perform the study in accordance with this protocol, will obtain consent and assent, and will report unanticipated problems involving risks to subjects or others in accordance with The Children's Hospital of Philadelphia IRB Policies and Procedures and all federal requirements. Collection, recording, and reporting of data will be accurate and will ensure the privacy, health, and welfare of research subjects during and after the study.

2 STUDY OBJECTIVES

Our research objective is to identify components of video messages that motivate behavior change.

2.1 Primary Objective (or Aim)

The primary objective is to compare intent to enroll in a research discovery site after seeing a video with an inclusion appeal versus one without such an appeal—focusing on parents and guardians who identify as African or African American.

---

[6] Jeannine S. Skinner et al., "The Impact of an Educational Video on Clinical Trial Enrollment and Knowledge in Ethnic Minorities: A Randomized Control Trial," *Frontiers in Public Health* 7 (2019): 104, https://doi.org/10.3389/fpubh.2019.00104.

[7] Joseph Petraglia, "The Importance of Being Authentic: Persuasion, Narration, and Dialogue in Health Communication and Education," *Health Communication* 24, no. 2 (March 2009): 176–85, https://doi.org/10.1080/10410230802676771.

[8] Melanie B. Tannenbaum et al., "Appealing to Fear: A Meta-Analysis of Fear Appeal Effectiveness and Theories," *Psychological Bulletin* 141, no. 6 (November 2015): 1178–1204, https://doi.org/10.1037/a0039729.

[9] Graton Aurélien and Mailliez Melody, "A Theory of Guilt Appeals: A Review Showing the Importance of Investigating Cognitive Processes as Mediators between Emotion and Behavior," *Behavioral Sciences (Basel, Switzerland)* 9, no. 12 (November 20, 2019): E117, https://doi.org/10.3390/bs9120117.

2.2 Secondary Objectives (or Aim)

The secondary objective of this study is to assess message effectiveness and targetedness of a video inviting African/African American parents to participate in clinical trials involving their children.

3 INVESTIGATIONAL PLAN

3.1 General Schema of Study Design

This project will include two phases, each of which is described below. The first phase of the study is designed to have members of the CHOP patient community review and evaluate the video and inclusion appeal message. Once the video has achieved a high level of perceived effectiveness, we will begin the second phase. The second phase of the study will be a 3-arm randomized controlled trial, focusing on parents of children cared for in CHOP primary care clinics who self-identify as African Americans proficient in verbal and written English, and with no history of prior trial participation.

3.1.1 Trial

The study will take place in primary care clinics throughout the CHOP ambulatory care system.

Because this pilot will assess the impact of a video, the first phase of the study will consist of work leading to videos used through the rest of the study. This phase will leverage work currently underway at the Children's Hospital of Philadelphia (CHOP) to construct a library of messages aimed at improving parents' knowledge about, and willingness to participate in, clinical research. Although some of these videos are constructed, none focus on the specific question that will be the focus of this research. We will work closely with a subset of CHOP's Research Family Partners (RFP), who will assist with all aspects of script development, tailoring to the diverse community served by CHOP, and evaluation plan. The RFP will help us to identify at least two patient families who identify as African American wo have participated in trials to interview and record for this project. The rough video will be validated by a sample of RFP members who identify as being from a historically marginalized group. We will primarily evaluate perceived message effectiveness, using the six-item survey developed by KC Davis and colleagues.[11] We will make iterative changes to the rough video until its perceived effectiveness averages 4.0 or greater on a 1 –5 scale. We will create a separate video segment for the inclusion appeal. This separate segment will be reviewed and approved by the RFP using a similar method before being edited into a copy of the video. Once the video and inclusion appeal messages are finalized (we anticipate this taking no more than 3 meetings), we will finalize the two videos and complete post-production to incorporate background music, improve color, audio balance and edit transitions.

Once the videos are created and validated, we will conduct a 3-arm randomized trial, focusing on parents with children cared for by CHOP who self-identify as African or African Americans proficient in verbal and written English, and with no history of prior trial participation. We will work with the RFP to identify the ideal settings for recruitment, which may include clinics, community settings, and online social media sites. We are currently

planning to invite participants to complete the study at a Penn or CHOP facility, rather than at home. Study participants will complete materials using an online survey completion and video display system (REDCap or Qualtrics). Each participant will complete questions about their age, gender identity, race/ethnicity, education attainment, and their child's age and gender identity. We will then ask a subset of questions from the NIH Health Information National Trends Survey to assess clinical trials knowledge. After these questions, all eligible participants will be randomly show neither one of two videos (with or without the inclusion appeal) followed by a short survey about perceived effectiveness of the message or taken directly to the final question. The final question will ask if they are willing to receive messages about potential clinical trials for their child. If they select yes, they will be taken to the enrollment screen for the registry above and will be given assistance by the research coordinator to help them complete the process if required.

Each participant will be paid $20 for participating in the study. The primary outcome will be intention to join a research registry called the Research Discovery Finder that notifies parents about research studies. We will measure attempts to join, rather than joining, in case the process of joining is too complicated or confusing. Based on data from two similar studies in adults[137] we will need approximately 30 completed surveys per group to have 80% power to detect a difference with a 0.05 significance level. Results will be reported using standard descriptive statistics. Between group differences in intent to enroll will be analyzed using chi-square analysis, with significance set at the 0.05 level. We will use a series of one-way between-group analyses of variance to determine the impact of the experimental video on dependent variables.

3.1.2 Allocation to Treatment Groups and Blinding

n/a

3.1.3 Study Duration, Enrollment and Number of Sites

The study duration per subject will be approximately 6 months for Phase 1 parents, and one visit for Phase 2 (including screening), with no planned follow-up.

3.1.4 Total Number of Study Sites/Total Number of Subjects Projected

The study will be conducted at one site in the Philadelphia area.

It is expected that approximately 100 subjects will be enrolled to produce 90 evaluable subjects. The study enrollment is planned to conclude in October 2022.

3.2 Study Population

Subjects that do not meet all of the below enrollment criteria may not be enrolled. Any violations of these criteria must be reported in accordance with IRB Policies and Procedures.

3.2.1 Inclusion Criteria

- English speaking (Phase 1 and 2)
- Self-identified African or African American (Phase 2 only)
- Not already enrolled in registry, no history of clinical trial participation (Phase 2 only)
- At least one child in their care age newborn to 13 (Phase 2 only)

3.2.2 Index/Case Subject Exclusion CriteriA

- Parents under the age of 18
- Parents with all children over the age of 13
- Non-English speakers
- Previously enrolled in CHOP's registry, history of clinical trial participation

3.2.3 Control Subject Inclusion Criteria

Same as 3.2.1

3.2.4 Control Subject Exclusion Criteria

Same as 3.2.2

4 STUDY PROCEDURES

4.1 Visit 1 (Phase 1)

- Welcome and introductions
- Participants will return signed informed consent (mailed to them before the visit.)
- Participants will see a presentation about the goals of the study and be invited to ask questions.
- Participants will see a first version of the video.
- Participants will complete a video effectiveness survey.
- Participants will discuss the video (open-ended, informed by the survey questions)

Subsequent Visits (Phase 1, up to three additional visits)

- Welcome and introduction
- Participants will see a presentation about the goals of the study and summary of how the video has been changed.
- Participants will see a revised version of the video.
- Participants will complete a video effectiveness survey.
- Participants will discuss the video (open-ended, informed by the survey questions)

4.1 Visit 1 (Phase 2)

Detailed description of study visit including all procedures.

Potential participants will be invited to enroll in the study based on scheduled information and self-identified race/ethnicity.

Upon arrival to clinic, potential participants will complete a brief screening form related to the inclusion criteria (English proficiency, able to watch a video or see an image, African/African American race). If inclusion criteria are met, they will receive informed consent form (with review and discussion) and be asked to sign it.

Participants will complete an online survey containing: (1) demographic questions (their age, gender identity, education attainment, and their child's age and gender identity); (2) clinical trials knowledge, using a subset of questions from the NIH Health Information National Trends survey

Participants will be randomly shown either: (1) one of two videos (with or without the inclusion appeal) followed by a short survey about perceived effectiveness of the message; or (2) a paragraph and image describing the CHOP Research Discovery Finder.

Participants will be asked if they are willing to receive messages about potential clinical trials for their child from the CHOP Research Discovery Finder. If they select yes, they will be taken to the enrollment screen for the registry above and will be given assistance by the research coordinator to help them complete the process if required.

Each participant will be paid $20 for participating in the study.

Subject Completion/Withdrawal

Subjects may withdraw from the study at any time without prejudice to their care. The Investigator or the Sponsor (if applicable) may also withdraw subjects who violate the study plan, or to protect the subject for reasons of safety or for administrative reasons. It will be documented whether or not each subject completes the study.

Early Termination Study Visit

Subjects who withdraw from the study after the study has begun and at least one question has been answered on the survey (out of time, lack of willingness or ability to continue, etc.) will be compensated as if the study had been completed.

Safety Evaluation

n/a

5 STATISTICAL CONSIDERATIONS

5.1 Primary Endpoint

The primary endpoint will be the difference in intent to enroll between the three groups.

5.2 Secondary Endpoints

A secondary endpoints will be the difference in message effectiveness between the video with versus without the inclusion appeal, evaluated using a 6-question survey adopted from KC Davis and colleagues.[11]

5.3 Statistical Methods

5.3.1 Baseline Data

Baseline and demographic characteristics will be summarized by standard descriptive summaries (e.g. means and standard deviations for continuous variables such as age and percentages for categorical variables such as gender).

5.3.2 Efficacy Analysis

For our assessment of differences between the three groups' intent to enroll, we will use a series of one-way between-group analyses of variance to determine the impact of the experimental video vs. control video vs. no video on enrollment intent.

5.4 Sample Size and Power

Based on data from two similar studies in adults we will need approximately 20 completed surveys per group to have 80% power to detect a difference with a 0.05 significance level. Because our plan is to conduct the study before or after scheduled clinic visits, where illness, weather and transportation may impact a family's ability to complete the study, we plan to enroll 90 patients to account for this attrition.

5.5 Interim Analysis

Interim analysis will not be necessary for this pilot study.

6 SAFETY MANAGEMENT

Since the study procedures are not greater than minimal risk, SAEs are not expected. If any unanticipated problems related to the research involving risks to subjects or others happen during the course of this study (including SAEs) they will be reported to the IRB in accordance with CHOP IRB SOP 408: Unanticipated Problems Involving Risks to Subjects. AEs that do not meet prompt reporting requirements will be summarized in narrative or other format and submitted to the IRB at the time of continuing review (if continuing reviews are required), or will be tracked and documented internally by the study team but not submitted to the IRB (if continuing reviews are not required).

6.1 Definition of an Adverse Event

An adverse event is any untoward medical occurrence in a subject who has received an intervention (drug, biologic, or other intervention). The occurrence does not necessarily have to have a causal relationship with the treatment. An AE can therefore be any unfavorable or unintended sign (including an abnormal laboratory finding, for example), symptom, or disease temporally associated with the use of a medicinal product, whether or not considered related to the medicinal product.

All AEs (including serious AEs) will be noted in the study records and on the case report form with a full description including the nature, date and time of onset, determination of non-serious versus serious, intensity (mild, moderate, severe), duration, causality, and outcome of the event.

6.2 Definition of a Serious Adverse Event (SAE)

An SAE is any adverse drug experience occurring at any dose that results in any of the following outcomes:

- death,
- a life-threatening event (at risk of death at the time of the event),
- requires inpatient hospitalization or prolongation of existing hospitalization,
- a persistent or significant disability/incapacity, or
- a congenital anomaly/birth defect in the offspring of a subject.

Important medical events that may not result in death, be life-threatening, or require hospitalization may be considered a serious adverse drug event when, based upon appropriate medical judgment, they may jeopardize the subject and may require medical or surgical intervention to prevent one of the outcomes listed in this definition.

A distinction should be drawn between serious and severe AEs. A severe AE is a major event of its type. A severe AE does not necessarily need to be considered serious. For example, nausea which persists for several hours may be considered severe nausea, but would not be an SAE. On the other hand, a stroke that results in only a limited degree of disability may be considered a mild stroke, but would be an SAE.

6.2.1 Relationship of SAE to study drug or other intervention

The relationship of each SAE to the study intervention should be characterized using one of the following terms in accordance with CHOP IRB Guidelines: definitely, probably, possibly, unlikely or unrelated.

6.3 IRB/IEC Notification of SAEs and Other Unanticipated Problems

The Investigator will promptly notify the IRB of all on-site unanticipated, serious Adverse Events that are related to the research activity. Other unanticipated problems related to the research involving risk to subjects or others will also be reported promptly. Written reports will be filed using the eIRB system and in accordance with the timeline below. External SAEs that are both unexpected and related to the study intervention will be reported promptly after the investigator receives the report.

Type of Unanticipated Problem	Initial Notification (Phone, Email, Fax)	Written Report
Internal (on-site) SAEs<br>Death or Life Threatening	24 hours	Within 2 calendar days

Internal (on-site) SAEs<br>All other SAEs	7 days	Within 7 business days
Unanticipated Problems Related to Research	7 days	Within 7 business days
**All other AEs**	**N/A**	**Brief Summary of important AEs may be reported at time of continuing review**

6.3.1 Follow-up report

If an SAE has not resolved at the time of the initial report and new information arises that changes the investigator's assessment of the event, a follow-up report including all relevant new or reassessed information (e.g., concomitant medication, medical history) should be submitted to the IRB. The investigator is responsible for ensuring that all SAE are followed until either resolved or stable.

7 STUDY ADMINISTRATION

7.1 Treatment Assignment Methods

7.1.1 Randomizations

One of three arms will be randomly presented to each participant through a specialized survey flow. Participants will then be administered one of three arms through a web-based randomizer. This data will be recorded and stored in Qualtrics.

7.1.2 Blinding

No blinding will be required.

7.1.3 Unblinding

N/A

7.2 Data Collection and Management

All data and records will be kept confidential and stored in Qualtrics

All data will be backed up in a password protected file in Box, and will be analyzed using Excel and R.

7.3 Confidentiality

All data and records generated during this study will be kept confidential in accordance with Institutional policies and HIPAA on subject privacy.

No identifiable data will be used for future study without first obtaining IRB approval or determination of exemption. The investigator will obtain a data use agreement between the provider (the PI) of the data and any recipient researchers (including others at CHOP) before sharing a limited dataset (PHI limited to dates and zip codes).

7.4 Regulatory and Ethical Considerations

7.4.1 Data and Safety Monitoring Plan

The CHOP PI will monitor and review the study progress, subject safety, and the accuracy and security of the emerging data.

7.4.2 Risk Assessment

This study, which does not directly involve children, and which consists only of surveys conducted during a pre-scheduled clinic visit, qualifies as a minimal risk study.

7.4.3 Potential Benefits of Trial Participation

This study does not confer direct benefits (other than compensation) to participants.

7.4.4 Risk-Benefit Assessment

This study which has essentially no risk and a very small monetary benefit is justifiably more benefit than risk.

7.5 Recruitment Strategy

Participants in the first phase of the study will be selected from families already signed up for the Research Family Partners program at CHOP. We are selecting no more than 9 parents from this group to advise us on study design, video development, message effectiveness and targetedness. Meetings will be using teleconferencing software.

Participants in the second phase will be invited to participate based on a review of the scheduled visits to the selected primary care sites each week. We will complete the study procedures in the waiting room or exam room before the visit (depending on the staffing and clinic flow, determined by asking the nurse manager each day we are in the clinic), and asking them to meet with us at the conclusion of the visit to complete the study if they are eligible and we were unable to complete the study initially. We do not anticipate requiring advertising.

7.6 Informed Consent/Assent and HIPAA Authorization

Members of the research team will review the study and obtain informed consent. No additional HIPAA authorization is required. Once a family member has agreed to participate, she will be given an iPad that displays a survey that already contains the patient's research ID. We will ask screening questions at that time. Parents/guardians who screen as eligible for the study will be invited to continue in the study after the visit. Surveys will be completed using researcher-provided iPads, and will be completed in the waiting room. Privacy will be assured through the use of headphones during the time the video is playing, and through the use of an iPad to collect the information.

7.6.1 Screening

We will complete screening in the clinic waiting room or exam room. The questions will be provided as a survey given to parents who are in the clinic and who are willing to be screened.

7.6.2 Main Study

A research coordinator (RC) will screen…

The RC will screen patients while awaiting the start of their visit and then check for their eligibility. Once it is determined that they are interested in participating in the study. We will consent them, review their eligibility, and use Qualtrics randomization tool to assign the participant to one of the three arms.

No HIPAA authorization form will be needed for this study.

8 CONSENT/HIPAA AUTHORIZATION PLAN FOR SUBJECTS WHO REACH AGE OF MAJORITY

n/a

8.1 Waiver of Consent

n/a

8.2 Waiver of Assent

Because the subjects of this research are parents and guardians, we will not require assent from their children.

8.3 Waiver of HIPAA Authorization

We request a waiver of HIPAA Authorization. The use or disclosure of protected health information involves no more than a minimal risk to the privacy of individuals (during the screening process, where we would see the age of the child, the identified race/ethnicity of the child, the language preference of the caregiver, and the name of the parents/guardians. All identifiers will be destroyed at the conclusion of data collection and payment.

8.4 Payment to Subjects/Families

8.4.1 Payments to subject for time, effort and inconvenience (i.e. compensation)

Each participant will be paid $20 for participating in the study. We will assume that the study will begin once they have started the survey

9 PUBLICATIONS

Only aggregate and unidentifiable data will be found in publications of this work.

REFERENCES

1. Hussain-Gambles M, Atkin K, Leese B. Why ethnic minority groups are under-represented in clinical trials: a review of the literature. *Health Soc Care Community*. 2004;12(5):382-388. doi:10.1111/j.1365-2524.2004.00507.x

2. Occa A, Morgan SE, Potter JE. Underrepresentation of Hispanics and Other Minorities in Clinical Trials: Recruiters' Perspectives. *J Racial Ethn Health Disparities*. 2018;5(2):322-332. doi:10.1007/s40615-017-0373-x

3. Green MC, Brock TC. The role of transportation in the persuasiveness of public narratives. *Journal of Personality and Social Psychology*. 2000;79(5):701-721. doi:10.1037/0022-3514.79.5.701

4. Shen F, Sheer VC, Li R. Impact of Narratives on Persuasion in Health Communication: A Meta-Analysis. *Journal of Advertising*. 2015;44(2):105-113. doi:10.1080/00913367.2015.1018467

5. Jimenez ME, Crabtree BF, Hudson SV, et al. Enhancing Reach Out and Read With a Video and Text Messages: A Randomized Trial in a Low-Income Predominantly Latino Sample. *Acad Pediatr*. 2021;21(6):968-976. doi:10.1016/j.acap.2021.02.011

6. Frew PM, Kriss JL, Chamberlain AT, et al. A randomized trial of maternal influenza immunization decision-making: A test of persuasive messaging models. *Hum Vaccin Immunother*. 2016;12(8):1989-1996. doi:10.1080/21645515.2016.1199309

7. Skinner JS, Fair AM, Holman AS, Boyer AP, Wilkins CH. The Impact of an Educational Video on Clinical Trial Enrollment and Knowledge in Ethnic Minorities: A Randomized Control Trial. *Front Public Health*. 2019;7:104. doi:10.3389/fpubh.2019.00104

8. Petraglia J. The importance of being authentic: persuasion, narration, and dialogue in health communication and education. *Health Commun*. 2009;24(2):176-185. doi:10.1080/10410230802676771

9. Tannenbaum MB, Hepler J, Zimmerman RS, et al. Appealing to fear: A meta-analysis of fear appeal effectiveness and theories. *Psychol Bull*. 2015;141(6):1178-1204. doi:10.1037/a0039729

10. Aurélien G, Melody M. A Theory of Guilt Appeals: A Review Showing the Importance of Investigating Cognitive Processes as Mediators between Emotion and Behavior. *Behav Sci (Basel)*. 2019;9(12):E117. doi:10.3390/bs9120117

11. Davis KC, Nonnemaker J, Duke J, Farrelly MC. Perceived effectiveness of cessation advertisements: the importance of audience reactions and practical implications for media campaign planning. *Health Commun*. 2013;28(5):461-472. doi:10.1080/10410236.2012.696535

12. Aaker JL, Brumbaugh AM, Grier SA. Nontarget Markets and Viewer Distinctiveness: The Impact of Target Marketing on Advertising Attitudes. *Journal of Consumer Psychology*. 2000;9(3):127-140. doi:10.1207/S15327663JCP0903_1

13. Winston S. Health Information National Trends Survey (HINTS.gov). *Med Ref Serv Q*. 2021;40(2):215-223. doi:10.1080/02763869.2021.1912575

14. Ma GX, Tan Y, Blakeney NC, et al. The impact of a community-based clinical trial educational intervention among underrepresented Chinese Americans. *Cancer Epidemiol Biomarkers Prev*. 2014;23(3):424-432. doi:10.1158/1055-9965.EPI-13-0773

APPENDIX

Append relevant information.
