# Supplementary material for: Culturally Tailored Messages and Trial Registry Enrollment: A Randomized Clinical Trial
Source: JAMA Netw Open. 2024 Nov 12;7(11):e2444229. doi: 10.1001/jamanetworkopen.2024.44229 (PMC11558476; doi:10.1001/jamanetworkopen.2024.44229)
Supplement: Supplement 2. — Data Sharing Statement [file jamanetwopen-e2444229-s002.pdf]

Data Sharing Statement

Johnson. Culturally Tailored Messages and Trial Registry Enrollment. *JAMA Netw Open*. Published November 12, 2024. doi:10.1001/jamanetworkopen.2024.44229

Data
Additional Information: Trial registration: Clinicaltrials.gov, registration number NCT06138145; URL https://clinicaltrials.gov/study/NCT06138145
Data available: No

Additional Information
Explanation for why data not available: Being used for another study related to the same topic, still under analysis.
